# IL-13 receptor α2 is a negative prognostic factor in human lung cancer and stimulates lung cancer growth in mice

**DOI:** 10.18632/oncotarget.5361

**Published:** 2015-09-21

**Authors:** Mian Xie, Xiao-jun Wu, Jin-jun Zhang, Chao-sheng He

**Affiliations:** ^1^ China State Key Laboratory of Respiratory Disease, The First Affiliated Hospital of Guangzhou Medical University, Guangzhou, China; ^2^ State Key Laboratory of Oncology in Southern China, Department of Colorectal Surgery, Sun Yat-sen University Cancer Center, Guangzhou, China; ^3^ Collaborative Innovation Center of Cancer Medicine, Guangzhou, China; ^4^ Department of Anesthesiology, The First Affiliated Hospital, Sun Yat-sen University, Guangzhou, China; ^5^ Department of Internal Medicine, Guangdong General Hospital, Guangzhou, China

**Keywords:** lung cancer, IL13Rα2, TAZ, PI3K

## Abstract

IL-13 receptor subunit alpha-2 (IL13Rα2) is associated with poor prognosis in some cancers. However, the role of IL13Rα2 in lung cancer remains unknown. We showed that IL13Rα2 overexpression was associated with late stages of disease progression and shorter disease-free survival (DFS) as well as overall survival (OS) in resected lung cancer patients. IL13Rα2 promoted the migration, invasion and anoikis resistance of lung cancer cells *in vitro*. Silencing of IL13Rα2 in lung cancer cells decreased invasion *in vitro* and lung metastasis *in vivo*. IL13Rα2 activated phosphatidylinositol 3 kinase (PI3K), Akt, and transcriptional coactivator with PDZ-binding motif (TAZ). Inhibition of PI3K attenuated activation of TAZ and its downstream target genes by IL13Rα2. We suggest that inhibition of IL13Rα2 is a potential therapeutic approach in lung cancer.

## INTRODUCTION

Chemokine-mediated inflammation participates in tumor growth, invasion, and metastasis [1]. The presence of proinflammatory molecules as interleukins (ILs) are typical features of cancer-related inflammation [2]. IL-13 is a proinflammatory, Th2-derived cytokine which is associated to different pathological conditions, such as asthma, autoimmune diseases, and ulcerative colitis [3]. IL-13 binds to two receptor subunits, IL-13 receptor subunit alpha-1 (IL13Rα1) and IL-13 receptor subunit alpha-2 (IL13Rα2). IL13Rα2 has been shown to be highly expressed in many tumor types, such as colon, glioblastoma, ovarian, head and neck, kidney, and mesothelioma, but not by most normal cells such as immune cells or endothelial cells [4–9]. IL13Rα2 is also associated with poor prognosis in human cancers and a target for cancer therapy [10–11]. IL-13 binding to IL13Rα2 increased tumor migration and invasion. Silencing of IL13Rα2 prolonged mice survival in mouse glioblastoma xenograft models [12]. IL13Rα2 participated in signal transduction, triggering the activation of several signaling proteins, such as MAPK and TGF-β1 [13–14]. However, little was known about the role of IL13Rα2 during lung cancer progression.

Toxins such as IL13-PE38QQR (the recombinant cytotoxin composed of IL-13 and a truncated form of pseudomonas aeruginosa exotoxin) have been designed to inhibit the IL-13 receptor. The phase III trial (PRECISE study) showed that IL13-PE38QQR mediated similar effects to Gliadel Wafer, a FDA approved drug. The trial failed to achieve its objective of superiority over Gliadel Wafer due to non-selective patients based on IL-13 receptor expression, catheter positioning, and poor drug distribution. The clinical toxicity of IL13-PE38QQR was likely because of non-selective binding of the toxin to the IL13Rα1/IL-4α type II receptor [15–19]. Rather than targeting the IL-13 receptor with a toxin, we have taken an alternative approach to specifically inhibit the expression of IL13Rα2 by using short hairpin RNA (shRNA).

In this study, we observed that overexpression of IL13Rα2 was associated with poor outcome of resected non-small cell lung cancer (NSCLC) patients. IL13Rα2 promoted lung cancer growth and invasion *in vitro* and *in vivo*. IL13Rα2 activated the phosphatidylinositol 3 kinase (PI3K), Akt, and transcriptional coactivator with PDZ-binding motif (TAZ).

## RESULTS

### IL13Rα2 is associated with poor prognosis in resected lung cancer patients

**Figure 1:**
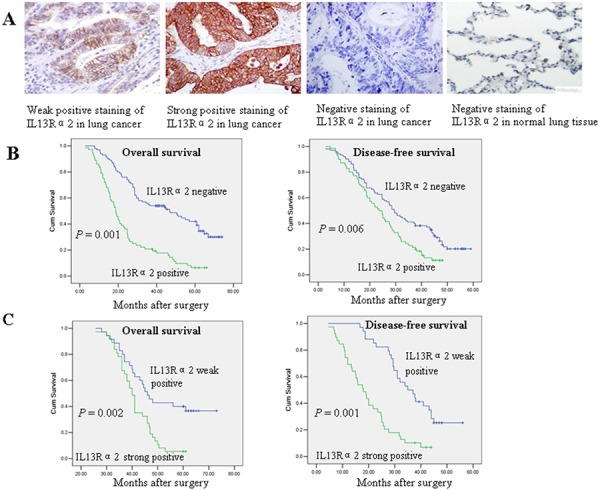
IL13Rα2 overexpression is associated with poor prognosis in resected lung cancer patients **A.** Immunohistochemistry staining of IL13Rα2 in lung cancer and normal lung tissues. Original magnification, × 200. **B.** Overall survival (OS) and disease-free survival (DFS) curves with IL13Rα2 negative (*H* score = 0) or positive expression (*H* score > 0) in resected lung cancer patients. **C.** OS and DFS curves among resected lung cancer patients with IL13Rα2 weak positive expression (*H* score 1 to 50) or strong positive expression (*H* score > 50).

To evaluate the prognostic significance of IL13Rα2 in lung cancer, IL13Rα2 expression was examined via immunohistochemistry (IHC) analysis for 181 resected NSCLC patients. IL13Rα2 expression was mainly associated to human lung cancer, while weak or no IL13Rα2 expression was found in adjacent normal lung tissues. Positive immunostaining for IL13Rα2 was mainly observed in the membrane of cancer cells. Representative images of weak, strong and negative IL13Rα2 staining are shown in Figure 1A. IL13Rα2 positive expression in tumor tissues was observed in 79 of 181 (43.6%) NSCLC patients. Relationships between IL13Rα2 expression and clinicopathological parameters were analyzed (Table 1). IL13Rα2 positive expression was more frequently detected in lung adenocarcinoma than the other histological types (*P* = 0.01). IL13Rα2 positive expression also correlated with poor tumor differentiation (*P* = 0.02), nodal status (*P* = 0.01) and TNM stage (*P* = 0.001). Survival analysis for resected NSCLC patients with or without IL13Rα2 expression was performed via the Kaplan-Meier method. All patients were included in the analysis. The median follow-up duration was 74.3 months. The median overall survival (OS) for patients with IL13Rα2 negative expression and positive expression were 55.9 months and 42.3 months, respectively. The median disease-free survival (DFS) for patients with IL13Rα2 negative expression and positive expression were 32.8 months and 23.1 months, respectively. Survival analysis showed a clear association with poor prognosis in terms of lower OS (*P* = 0.001) and DFS (*P* = 0.006) for patients with IL13Rα2 positive expression (Figure 1B). In subgroup analysis, the median OS was longer in patients with IL13Rα2 weak positive expression (39.7 months) than in patients with IL13Rα2 strong positive expression (27.3 months) (*P* = 0.002). Similarly, the median DFS was longer in patients with IL13Rα2 weak positive expression (30.7 months) than in patients with IL13Rα2 strong positive expression (18.9 months) (*P* = 0.001) (Figure 1C). The results suggest that IL13Rα2 is a negative prognostic factor in resected NSCLC patients.

**Table 1:** Relationship between IL13Rα2 expression and clinicopathological parameters

Characteristics	IL13Rα2 expression			Total (*n* = 181)	*P* value
	Positive		Negative (*n* = 102)		
	Weak (*n* = 45)	Strong (*n* = 34)			
Age (yr)					0.08
< 65	26	18	57	101	
≥ 65	19	16	45	80	
Gender					0.06
Male	30	21	66	117	
Female	15	13	36	64	
Histology					0.01
Adenocarcinoma	28	24	62	114	
Squamous carcinoma	15	10	36	61	
Others	2	0	4	6	
Tumor differentiation					0.02
Well/moderate	23	10	80	113	
Poor	22	24	22	68	
Nodal status					0.01
N0	8	3	48	59	
N1/N2	37	31	54	122	
TNM stage					0.001
I	6	2	57	65	
II/IIIA	39	32	45	116	

### IL13Rα2 promotes cell proliferation, invasion, migration and anoikis resistance in lung cancer cells

**Figure 2:**
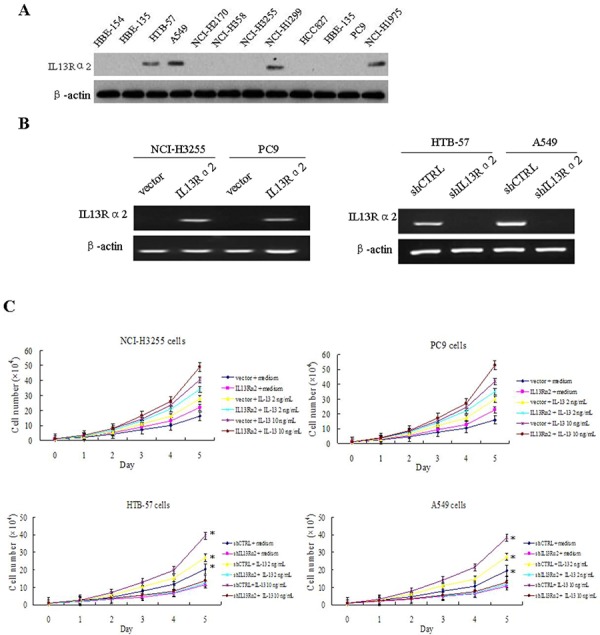

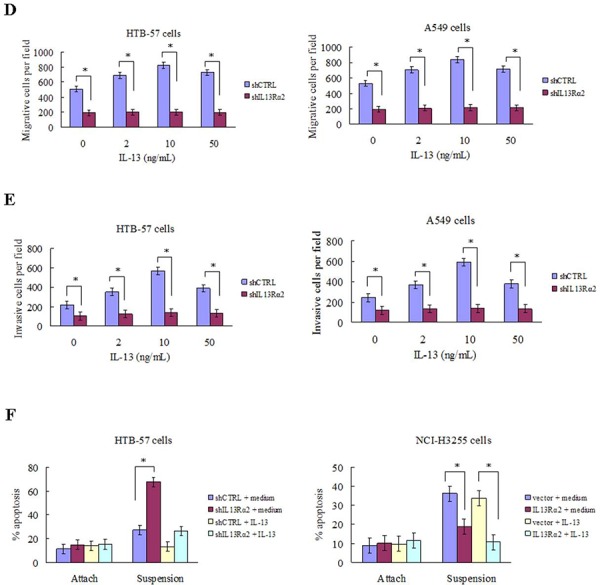
IL13Rα2 promotes proliferation, invasion, migration and anoikis resistance in lung cancer cells **A.** The protein expression level of IL13Rα2 in lung cancer cells and normal lung epithelial cells were measured using western blotting assay. **B.** HTB-57 and A549 cells were transfected with control shRNA (shCTRL) or IL13Rα2 shRNA (shIL13Rα2). NCI-H3255 and PC9 cells were transfected with IL13Rα2 or control vector. Transfection efficiency was evaluated by RT-PCR analysis. **C.** Proliferation assays were performed by CCK-8 kit in IL13Rα2-transfected and IL13Rα2-silenced lung cancer cells pretreated with or without IL-13. *P < 0.05 was considered statistically significant when compared with control cells. **D-E.** Transwell migration and invasion assays were conducted. HTB-57 and A549 cells transfected with shIL13Rα2 or shCTRL were treated in presence of medium alone or with IL-13. The cell numbers at the bottom are the means ± SD of the counts in eight random fields for three independent experiments. *P < 0.05. **F.** IL13Rα2 transfected NCI-H3255 cells and IL13Rα2 silenced HTB-57 cells were cultured on tissue culture plates (attach) or on poly-HEMA pre-coated plates (suspension) in presence of IL-13. After 72 h, cells were collected, stained with PE Annexin V and analyzed by flow cytometry. Experiments were done in duplicate. *P < 0.05.

We examined the expression level of IL13Rα2 using western blotting in a panel of human lung cancer cells and normal lung epithelial cell lines. The results indicated that the protein expression of IL13Rα2 was higher in HTB-57, NCI-H1975, NCI-H1299 and A549 cells compared with the others lung cancer cells and normal lung epithelial cells (Figure 2A). HTB-57 and A549 cells were transfected with IL13Rα2 shRNA (shIL13Rα2) or control shRNA (shCTRL). NCI-H3255 and PC9 cells were transfected with IL13Rα2 or control vector. Expression of IL13Rα2 was confirmed by reverse transcriptase-polymerase chain reaction (RT-PCR) assay (Figure 2B). IL13Rα2 transfection in NCI-H3255 and PC9 cells increased cell proliferation in response of IL-13 compared with control cells. Addition of 10 ng/mL IL-13 in NCI-H3255 and PC9 cells enhanced proliferation more dramatically than that of 2 ng/mL IL-13. However, IL13Rα2 silencing in HTB-57 and A549 cells resulted in a significantly inhibited cell growth rate. Addition of IL-13 increased cell growth in control cells at the concentration of 10 ng/mL (*P* < 0.05), but not in the silenced cells (Figure 2C). Next we studied the effects exerted by IL13Rα2 on tumor cell migration and invasion. Compared with the control cells, knockdown of IL13Rα2 significantly inhibited the abilities of migration (*P* < 0.05) and invasion (*P* < 0.05) in HTB-57 and A549 cells. Addition of IL-13 caused a significant increase of invasion in the control HTB-57 and A549 cells, with an optimum at 10 ng/mL. In contrast, IL13Rα2 silenced cells were insensitive to IL-13, similar to basal levels (Figure 2D–E). These results indicated that IL13Rα2 increased lung cancer cell growth, migration and invasion. Anoikis is a programmed cell death process that is induced upon cell detachment from the extracellular matrix (ECM) and anoikis resistance is a critical mechanism during tumor progression and metastasis [20]. Ectopic expression of IL13Rα2 significantly attenuated anoikis of NCI-H3255 cells at the presence of IL-13 in suspension, while knockdown of IL13Rα2 showed increased anoikis in HTB-57 cells (*P* < 0.05) (Figure 2F).

### IL13Rα2 promotes tumor growth and lung metastasis *in vivo*

**Figure 3:**
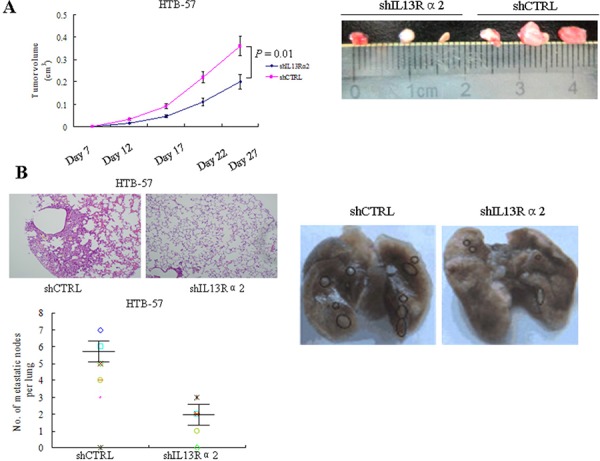
IL13Rα2 promotes tumor growth and lung metastasis *in vivo* **A.** Nude mice were subcutaneously injected with 1.5 × 10^6^ HTB-57 cells stably transfected with shIL13Rα2 or shCTRL. Tumor volumes were measured every five days after tumor cell implantation for 28 days. Data are presented as the means ± SD and compared between groups using the *t*-test. *P* < 0.05 was considered statistically significant between the two groups. **B.** Representative images of lung metastases of HTB-57 cells. Nude mice were tail vein injected with 1.5 × 10^6^ HTB-57 cells stably transfected with shIL13Rα2 or shCTRL. Numbers of lung metastases in nude mice were counted after 8 weeks (data are presented as the means ± SD; *n* = 8 mice per group).

We next investigated the effects of IL13Rα2 on tumor growth and metastasis *in vivo*. We subcutaneously implanted HTB-57 cells transfected with shIL13Rα2 or shCTRL into the nude mice. Consistent with the results obtained from the *in vitro* assays, silencing of IL13Rα2 inhibited xenograft tumor growth (*P* = 0.01) (Figure 3A). For an *in vivo* metastasis assay, HTB-57 cells transfected with shIL13Rα2 or shCTRL were injected into the tail vein of the nude mice. Mice were sacrificed after 8 weeks. Primary and metastatic tumor tissues were pathologically examined, and the number of metastasized lung cancer nodules was compared between the two groups of nude mice. The average number of lung metastases in mice inoculated with HTB-57 cells with shIL13Rα2 was 1.93 ± 1.1 per mouse while the number of lung metastases in the control group was 5.8 ± 1.3 per mouse (*P* = 0.001) (Figure 3B). These results demonstrated that IL13Rα2 promotes tumor growth and lung metastasis *in vivo*.

### IL13Rα2 enhances the PI3K-TAZ pathway in lung cancer cells

**Figure 4:**
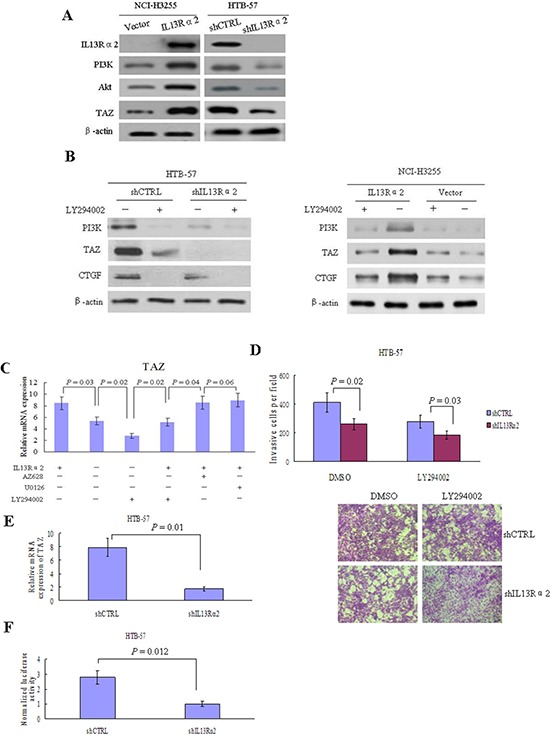
IL13Rα2 activates TAZ through PI3K in lung cancer **A.** HTB-57 cells were transfected with shIL13Rα2 and NCI-H3255 cells were transfected with IL13Rα2, respectively. The expressions of IL13Rα2, PI3K, Akt and TAZ were examined using western blotting. **B.** IL13Rα2 transfected NCI-H3255 cells and IL13Rα2 silenced HTB-57 cells were treated with 10 μM LY294002 or DMSO for 48 hours. The protein expression levels of PI3K, TAZ, and CTGF were evaluated using western blotting. **C.** AZ628 (2 μM), U0126 (10 μM), or LY294002 (10 μM) was added respectively into the cell culture medium of HTB-57 cells transfeced with IL13Rα2 or control vector. After 24 hours, qPCR was used to determine the mRNA expression level of TAZ. Data are presented as the means ± SD and groups were compared using the *t*-test. *P* < 0.05 was considered statistically significant between two groups. **D.** HTB-57 cells transfected with shIL13Rα2 or shCTRL were treated with 10 μM LY294002 or DMSO for 48 hours. Cell invasion was measured by matrigel invasion assay. **E.** The relative mRNA expression of TAZ was measured by qPCR in HTB-57 cells transfected with shIL13Rα2 or shCTRL. **F.** Luciferase reporter gene assay results. Luciferase activities were measured after pGL3-promoter-TAZ was transfected into IL13Rα2 silenced HTB-57 cells or control cells.

**Figure 5:**
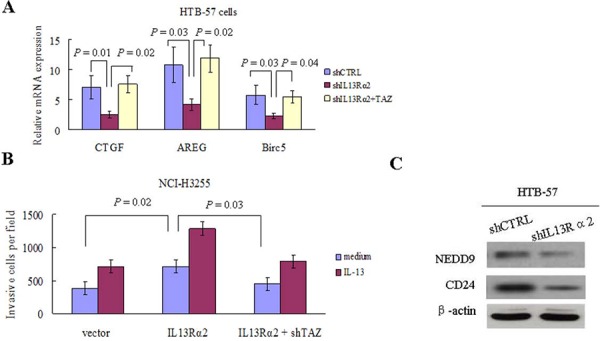
IL13Rα2 promotes invasion and metastasis through TAZ and its downstream target genes **A.** qPCR was used to determine mRNA expression levels of CTGF, AREG and Birc5 in HTB-57 cells transfected with shCTRL, shIL13Rα2 alone or in combination with TAZ. **B.** Matrigel invasion assay were performed in NCI-H3255 cells transfected with IL13Rα2 or IL13Rα2 combined with shTAZ in presence of medium or IL-13. **C.** The expressions of NEDD9 and CD24 in HTB-57 cells transfected with shIL13Rα2 or shCTRL were examined using western blotting assay.

Silencing of IL13Rα2 in HTB-57 cells caused a marked inhibition of PI3K, Akt, and TAZ. Conversely, IL13Rα2 transfection into NCI-H3255 cells, which lack of endogenous IL-13Rα2 expression, increased the expressions of PI3K, Akt, and TAZ (Figure 4A). The TAZ protein level is elevated in tumor cells via increased PI3K signaling, which raises the possibility that IL13Rα2 activates the TAZ pathway through PI3K [21, 22]. As shown in Figure 4B, IL13Rα2 silencing significantly decreased the protein expressions of TAZ and its downstream target gene CTGF in IL13Rα2 high-expression HTB-57 cells. The addition of LY294002 further attenuated the inhibition of TAZ and CTGF by IL13Rα2 shRNA. LY294002 alone exerted no obvious inhibitory effects on the TAZ pathway in IL13Rα2 low-expression NCI-H3255 cells. However, LY294002 largely abolished the activating effects of IL13Rα2 on TAZ and CTGF in NCI-H3255 cells. Real-time reverse transcription-PCR (qPCR) assay indicated that IL13Rα2 transfection significantly increased mRNA expression of TAZ in HTB-57 cells (*P* = 0.03), while PI3K inhibitor LY294002 significantly inhibited the mRNA expression of TAZ which was enhanced by IL13Rα2 transfection (*P* = 0.04). However, neither Raf1 inhibitor AZ628 nor MEK1/2 inhibitor U0126 had any effect on TAZ expression (Figure 4C). LY294002 decreased the invasive ability in IL13Rα2 silenced HTB-57 cells (Figure 4D). qPCR assay revealed that the relative mRNA expression level of TAZ was significantly lower in IL13Rα2 silenced HTB-57 cells than control cells (*P* = 0.01) (Figure 4E). The activity of the TAZ promoter was significantly inhibited by IL13Rα2 shRNA, as shown with a dual luciferase reporter assay (*P* = 0.012) (Figure 4F). IL13Rα2 shRNA significantly decreased the mRNA levels of the TAZ downstream target genes CTGF (*P* = 0.01), AREG (*P* = 0.03) and Birc5 (*P* = 0.03). Meanwhile, the addition of TAZ transfection increased the mRNA expression of these TAZ downstream genes (*P* < 0.05) (Figure 5A). Moreover, TAZ silencing inhibited the invasive ability of NCI-H3255 cells transfected with IL13Rα2 (*P* = 0.03) (Figure 5B). Expression profiling in human lung cancer cell lines has identified NEDD9 and CD24 as metastasis-promoting genes [22]. Our study showed that IL13Rα2 shRNA suppressed the expressions of NEDD9 and CD24 via a western blotting assay (Figure 5C). Collectively, these data indicate that IL13Rα2 promotes lung cancer progression through PI3K-TAZ pathway.

## DISCUSSION

Here, we have described an important role for IL13Rα2 in lung cancer growth, invasion and metastasis. The conclusion was obtained from the following observations: (i) IL13Rα2 increased lung cancer cells growth and anoikis resistance. (ii) IL13Rα2 silencing suppressed lung cancer growth, invasion and metastasis. (iii) IL13Rα2 overexpression was more frequently detected in lung adenocarcinoma and associated with poor prognosis in resected lung cancer patients. Our work is consistent with a recent study which indicated IL13Rα2 could be a biomarker for lung cancer especially lung adenocarcinoma [23, 24].

In our model, IL13Rα2 signaling increased tumor invasion through PI3K activation, as PI3K inhibitor LY294002 blocked the effect of IL13Rα2 on lung cancer invasion. TAZ activation was found on IL13Rα2 transfected lung cancer cells and was associated with lung cancer invasion. CTGF, AREG and Birc5 are direct targets of TAZ [25]. CTGF promotes melanoma cell invasion and migration, and anti-CTGF antibodies significantly inhibit the progression of metastatic melanoma [26]. AREG induces growth signals, invasion and metastasis [27]. Birc5 inhibits apoptosis and plays an essential role in tumorigenesis [28]. Our results demonstrated that IL13Rα2 promoted invasion by activating TAZ and its downstream targets CTGF, AREG and Birc5. Additionally, we found that IL13Rα2 increased the expression level of the metastasis-promoting genes NEDD9 and CD24. Taken together, these data strongly suggest that IL13Rα2 promote lung cancer growth and metastasis. Therefore, further study should focus on IL13Rα2-targeted therapeutic strategies.

In this study, we elucidated the role for PI3K in the signal transduction of IL13Rα2 for the invasion of lung cancer cells. The binding of IL-13 triggers the recruitment of several molecules to IL13Rα2, such as PI3K and TAZ. Huang et al observed that the TAZ protein level was elevated in tumor cells with high PI3K signaling. TAZ was the growth-promoting signal downstream of the PI3K. When PI3K was activated, TAZ protein levels was accumulated, therefore contributing to the mitogenic activity of the PI3K pathway [22]. TAZ is one of the downstream effectors of the Hippo pathway and has recently been identified as an oncogene [29]. Our previous study revealed that TAZ expression was a prognostic indicator of poorer survival for resected lung cancer patients [25]. We verified that IL13Rα2 activated TAZ to promote lung cancer invasion *in vitro*. We further demonstrated that IL13Rα2 increased PI3K and TAZ expression in lung cancer cells. The PI3K inhibitor LY294002 significantly inhibited the TAZ expression which was activated by IL13Rα2. However, neither the Raf1 inhibitor AZ628 nor the MEK inhibitor U0126 had any effect on TAZ. Thus, IL13Rα2 increased TAZ expression via the PI3K/Akt pathway rather than the Raf1 or MEK pathways. Together, these results indicate that IL13Rα2 signaling requires PI3K to activate TAZ in lung cancer.

Our study showed a clear association between IL13Rα2 overexpression and poor survival in resected NSCLC patients. This worse prognosis could be attributed to the increased invasive and metastatic ability of IL13Rα2 in lung cancer cells. In conclusion, we reveal that IL13Rα2 promotes lung cancer growth, invasion and metastasis via the activating PI3K-TAZ pathway. Thus, inhibition of IL13Rα2 is a potential therapeutic approach in lung cancer.

## MATERIALS AND METHODS

### Cell lines, antibody and reagents

HBE-135, HBE-154, PC9, NCI-H1975, A549, HTB-57, NCI-H2170, NCI-H1299, NCI-H358, NCI-H3255, NCI-H1838, and HCC827 cell lines were purchased from the American Type Culture Collection (Manassas, VA). These cell lines were routinely cultured in RPMI-1640 supplemented with 10% fetal calf serum at 37°C in a 5% CO_2_ atmosphere. The antibodies used for western blotting were purchased as follows: IL13Rα2 from Abcam Inc; PI3K, TAZ, NEDD9, CD24, CTGF and Akt from Santa Cruz Biotechnology. U0126, LY294002, and AZ628 were purchased from Cell Signaling Inc. IL-13 was purchased from PeproTech (California, USA).

### Reverse transcriptase-polymerase chain reaction (RT-PCR)

RNA was isolated using the Trizol Plus kit (TaKaRa, Japan). First-strand cDNA synthesis was performed using Invitrogen kit. Subsequent PCR reactions were performed using an RNA-PCR kit (Takara, Japan). β-actin was used as an internal control.

### Real-time reverse transcription-PCR (qPCR) analysis

cDNA was synthesized as described above. qPCR analysis was performed using All-in-One™ qPCR Mix (GeneCopoeia, USA) according to the manufacturer's protocol. Relative changes in transcript levels were determined via β-actin normalization of mRNA levels.

### Cell transfection

The TAZ shRNA (shTAZ) plasmid, IL13Rα2 shRNA (shIL13Rα2), and control shRNA (shCTRL) were purchased from Santa Cruz Biotechnology. A pcDNA3.1/HisC plasmid (Invitrogen, Carlsbad, CA) was used to construct the TAZ plasmid. Recombinant IL13Rα2 plasmid (pET-28a-IL13Rα2) and the control vector were kindly offered by Wenzhou Medical College [30]. Cells were stably transfected with control vector, TAZ, IL13Rα2, shIL13Rα2 or shTAZ and maintained under geneticin (G418, Invitrogen) selection.

### Dual luciferase reporter assay

Cells were transfected with the indicated luciferase reporter. Relative Firefly luciferase activity to TK Renilla (internal control) was measured with dual-luciferase reporter assay system (Promega, USA) in an automatic microplate reader (Thermo, USA).

### Matrigel invasion and migration assay

Matrigel (BD Biosciences, USA) was applied to the upper chamber. Cells were trypsinized and seeded at 5 × 10^4^ cells per insert in 100 μL serum-free DMEM medium. Inserts were placed in 600 μL DMEM medium with 10% FBS. The cells were incubated for 24 h with 5% CO_2_ humidified atmosphere. Then the cells on the upper surface of the filters were completely removed by wiping with a cotton swab. The inserts were fixed in 4% polyfluoroalkoxy (PFA) for 30 min and stained with crystal violet staining solution (Beijing Zhongshan Biotechnology, China). The images were taken at 200× magnification with an Olympus microscope (Olympus Corporation, Japan). For invasion assay, the numbers of cells attached to the other side of the insert were counted under a light microscope in 8 random fields at a magnification of × 200. For migration assay, after photographed, the cells migrated to the underside were eluted by 33% acetic acid, and the O.D. values of absorbance were measured by VarioskanFlash (Thermo, USA) at 570 nm. The data shown were representatives of three independent experiments.

### Western blotting assay

Total protein was isolated from cells using cell extraction buffer (Biosource, Camarillo, CA) supplemented with protease and phosphatase inhibitors. Protein concentrations were measured using a BCA Protein Assay kit (Pierce, Rockford, IL). Antibodies against IL13Rα2, TAZ, PI3K, Akt, CTGF, CD24, NEDD9 and β-actin were used as primary antibodies. The blots were incubated with a horseradish peroxidase-conjugated goat anti-rabbit antibody (Santa Cruz Biotechnology). Proteins were visualized with electrochemical luminescence (GE Healthcare, CA).

### Cell proliferation assay

Cells (1 × 10^4^/well) were transferred into six-well plates for five days. CCK-8 solution was then added and incubated for 1 hour, and cell proliferation was evaluated every 24 h using a CCK8 solution kit. Cell viability was determined via scanning with a microplate reader at 450 nm.

### Anoikis assay

Anoikis assay were conducted as described previously [31]. Cells were cultured in attachment or suspension. For suspension condition, cells were seeded in poly-HEMA (Sigma-Aldrich; St. Louis, MO, USA) pre-coated 6-well plates at 37°C for 48 h. After 72 h, cells were trypsinized, stained with PE Annexin V and analyzed by flow cytometry using the PE Annexin V Apoptosis Detection kit (BD Biosciences). Data were collected and analyzed on a BD FACSCanto using FACSDiva software.

### Immunohistochemistry (IHC) analysis

IL13Rα2 expression was studied immunohistochemically in 181 consecutive patients with resected non-small cell lung cancer (NSCLC). The study was approved by the Ethics Committee of the First Affiliated Hospital of Guangzhou Medical University. Written informed consents were obtained from all subjects. None of the patients received radiotherapy or chemotherapy prior to operation. IHC analysis was performed as described previously [25]. The IL13Rα2 antibody used for IHC was purchased from Abcam Inc. Membrane immunostaining in tumor cells was considered positive. Tissue was scored (*H* score) based on the total percentage of positive cells and intensity of the staining (1+, 2+, or 3+), where *H* = (% 1 + × 1) + (% 2+ × 2) + (% 3 + × 3). The sample was considered negative if *H* = 0 and positive if *H* was more than 0; positive samples were also categorized as weak if *H* = 1 to 50 and strong if *H* was more than 50. A minimum of 100 cells were evaluated to calculate the *H* score.

### *In vivo* animal models

Four- to six-week-old female Balb/c athymic (nu+/nu+) mice were purchased from Shanghai Slac Laboratory Animal Co. Ltd. (China). The research protocol was approved by the First Affiliated Hospital of Guangzhou Medical University. For the xenograft tumorigenicity assay, 1.5 × 10^6^ lung cancer cells resuspended in 200 μL of matrigel (BD Biosciences, USA) were subcutaneously injected into the right flank of nude mice. Tumor volumes were measured every 5 days for 4 weeks. Tumor volume was determined using the formula π/6 × larger diameter × (smaller diameter). For the *in vivo* metastasis assay, cells (1.5 × 10^6^ in 0.1 mL PBS) were injected into nude mice via the tail vein. Mice were sacrificed after 8 weeks. Primary and metastatic tumor tissues were removed and confirmed, measured and embedded in 10% paraffin. Each tissue was subjected to H&E staining for histological examination.

### Statistical analysis

Data are presented as the mean ± standard deviations (SD). All statistical analyses were performed using the SPSS 16.0 software package. Comparisons between groups were evaluated using Student's *t*-test. Survival curves were assessed via the Kaplan-Meier method and compared with the log-rank test. A two-sided value of *P* < 0.05 was considered statistically significant. Disease-free survival (DFS) and overall survival (OS) were defined as the time from the date of surgery to the date of regional recurrence or distant metastasis and death or final clinical follow-up, respectively.
